# Whether vitamin D was associated with clinical outcome after IVF/ICSI: a systematic review and meta-analysis

**DOI:** 10.1186/s12958-018-0324-3

**Published:** 2018-02-09

**Authors:** Jing Zhao, Xi Huang, Bin Xu, Yi Yan, Qiong Zhang, Yanping Li

**Affiliations:** Reproductive Medicine Center, Xiangya Hospital, Central South University, 87 Xiang Ya Road, Changsha, Hunan 410008 People’s Republic of China

**Keywords:** Vitamin D, Clinical pregnancy, Live birth, Assisted reproduction technology

## Abstract

**Background:**

There exist contradictive views on whether the vitamin D has association with clinical outcome of in vitro fertilization (IVF) and/or intracytoplasmic sperm injection (ICSI). The present meta-analysis aim to establish whether vitamin D was associated with clinical outcomes of IVF/ICSI.

**Methods:**

MEDLINE, Google Scholar and the Cochrane Library from database inception to March 2017 were searched. Clinical studies, which evaluated the association of vitamin D level and the clinical outcomes after IVF/ICSI, were included. The Main Outcome Measures were clinical pregnancy, ongoing pregnancy, and live birth.

**Results:**

In the analysis of clinical pregnancy, 9 cohort studies were included. Of which, 2 studies and 3 studies were identified in analyzing ongoing pregnancy and live birth, respectively. Meta-analysis showed trends toward lower clinical pregnancy [RR 0.91, (95% CI 0.77–1.07)] and higher ongoing pregnancy [RR 1.06, (95% CI 0.95–1.19)] for women with deficient level of vitamin D. The probability of live birth for women with deficient level of vitamin D was significantly lower than cases with sufficient level of vitamin D [RR 0.74, (95% CI 0.58–0.90)].

**Conclusions:**

Deficient vitamin D was associated with decreased probability of live birth after IVF/ICSI. So vitamin D should be supplied to women with deficient level vitamin D.

**Electronic supplementary material:**

The online version of this article (10.1186/s12958-018-0324-3) contains supplementary material, which is available to authorized users.

## Background

As a steroid hormone, Vitamin D exerts effect by binding with vitamin D receptor, which is a member of the nuclear receptors families [1, 2]. It was established that many tissues have vitamin D receptor, such as ovarian, endometrium, fallopian tube epithelial cells, placenta, decidual cells, hypothalamus, and pituitary [3–6]. Vitamin D plays critical role in the system of female reproduction, such as oocyte development, production of anti-Mullerian (AMH), ovarian steroidogrnrsis, endometrial receptivity, et al. It was supposed that vitamin D is associated with clinical pregnancy outcome of IVF/ICSI cycles.

So far, there were myriads of studies evaluating the association between vitamin D level and the clinical outcomes after ART. Some studies have indicated that deficient vitamin D was associated with pregnancy rate [7–13]. In contrast, some researchers found no association between vitamin D statuses with outcomes after IVF/ICSI cycles [14–18].

At present, two meta-analysis studies have evaluated the association between vitamin D level and outcomes of IVF/ICSI. One was done by Vanni et al., including three studies with 353 women. The study only compared the one outcome that is--clinical pregnancy rate, and found that deficient vitamin D was associated with lower probability of clinical pregnancy without statistically significance compared with vitamin D sufficient women [19]. The other meta-analysis, including five studies with 1238 women, compared both clinical pregnancy rate and live birth rate. The results displayed that deficient vitamin D was uncorrelated with clinical pregnancy rate, but related to decreased probability of live birth [20]. Although the design of the previous meta-analysis was suitable, the samples size of some studies was small.

So, it is necessary to conduct a systematic review and meta-analysis including more suitable studies to evaluated further whether deficient level vitamin D have effect on clinical pregnancy, ongoing pregnancy, and live birth after IVF/ICSI.

## Methods

### Identification of the literature

MEDLINE, Google Scholar and the Cochrane Library from inception until March 2017 were searched. The terms used to search literatures were as follows: one including terms on vitamin D (vitamin D, 25-hydroxy vitamin D, 25(OH) D, 1,25-(OH)_2_D), the other one including terms on reproductive techniques (in vitro fertilization, IVF, intracytoplasmic sperm injection, ICSI, assisted reproduction technologies, ART). Related citations were generated by combining the above terms with “AND”. Only papers fully published in English were covered. These included papers were reviewed by two investigators independently, and group discussion was needed if there was any discrepancies.

### Study selection and data extraction

Studies evaluated the association between serum vitamin D levels and clinical outcomes after ART were selected. The primary outcomes of interest were clinical pregnancy and/or ongoing pregnancy, and /or live birth. For studies to be eligible, outcome data (with pregnancy rate and/or ongoing pregnancy rate and/or live birth rate in women with deficient or sufficient vitamin D levels) were extracted in 2 × 2 tables. We also recorded the study design, treatment protocol, threshold for deficient vitamin D, number of pregnant / live birth cycles with deficient or sufficient vitamin D levels. Newcastle–Ottawa Quality Assessment Scales was used to evaluate the quality of the observational studies [21]. Two reviewers completed the quality assessment, Group discussion was needed when there was any disagreements about inclusion.

### Statistical analysis

We applied the meta-analysis method to complete the comparisons of study-by-study with the risk ratios (RRs) of the individual 2 × 2 tables. Forest plots were used to evaluate the heterogeneity of the included studies and the l^2^ value quantified heterogeneity between studies. Mantel- Haenszel Random effect models were implied according to the heterogeneity to calculate an overall RR and its 95% CI. A *P*-value of 0.10 rather than conventional level of 0.05 was considered to have statistical significance because of the low power of the X^2^ test for heterogeneity when studies have small sample size. The meta-analysis was conducted with RevMan 5.0 (Cochrane Collaboration, Oxford, UK).

## Results

### Studies selection and characteristics

**Fig. 1:**
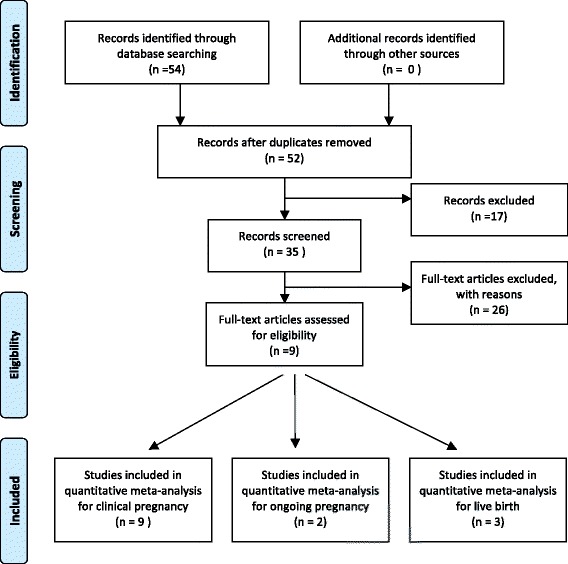
Flow chart showing study selection process

The search strategy yielded 52 citations, 17 of which are excluded after reviewing the titles and abstracts. Five of the 35 remaining publications were not suitable because either pregnancy or live birth data were not reported, and three papers were excluded because a 2 × 2 table was not extracted from the results. The remained 18 papers were reviews, meta-analysis or animal experiments. (Fig. 1).

The total number of eligible trails included in the present study was 9 (three IVF study, one ICSI study, three IVF/ICSI studies, and two oocyte donation studies) comprising 2254 couples reported clinical pregnancy rate after IVF/ICSI with 1112 pregnant cycles. 2 papers involved 784 couples also reported ongoing pregnancy rate and 3 studies involved 655 pregnant women with 238 live birth cycles.

The characteristics of included studies were depicted in Table 1. Of these 9 studies, 5 were retrospective studies and 4 were prospective studies. All the studies evaluated the vitamin D level in the serum, one study by Firouzabadi et al. [14] also examined the follicular fluid. The threshold for deficient vitamin D was 20 ng/ml in seven studies, 10 ng/ml in one study and 75 nmol/l in another study.

**Table 1:** Characteristic of studies included in our meta-analysis

Studies	Country	Study design	Protocol	Sample size	The time of sample collection	Vd grouping criteria	Outcome measures	Main result	Main confounding factors
Anifandis et al. 2010 [8]	Greece	Retrospective cohort study	Short agonist protocol IVF/ICIS	101	During oocyte retrieval	< 20 ng/ml; 20.1-30 ng/ml; > 30 ng/ml	Clinical pregnancy rate (CPR)	Higher FF levels of 25(OH)D are associated with lower FF glucose levels and with lower CPR	Age BMI Oocytes retrieved (n)
Rudick et al. 2012 [10]	USA	Retrospective cohort study	Long agonist Flare-up GnRH-ant IVF/ICSI	188	After hCG administration	< 20 ng/ml; 20-30 ng/ml; > 30 ng/ml	ou	Opposite relation between 25(OH)D levels and IVF outcomes by race	Age BMI Embryos transferred (n, quality) Poor ovarian reserve
Firouzabadi et al. 2013 [14]	Iran	Prospective cohort study	Long GnRH-a protocol IVF	221	On the day of ovum pick-up	< 10 ng/ml 10-29 ng/ml 30-100 ng/ml	CPR	No significant association between FF or serum levels of 25(OH)D and CPR	None
Garbedian et al. 2013 [9]	USA	Prospective cohort study	Long GnRH-a Flare-up GnRH-ant IVF	162	Before oocyte retrieval	< 75 nmol/l ≥75 nmol/l	CPR	High vitamin D level was related to higher CPR	Age BMI Day 5[v. day3] embryo transfer
Fabris et al. 2014 [17]	Spain	Retrospective cohort study	Egg donation recipients	267	After 2 weeks of HRT	< 20 ng/ml; 20-30 ng/ml; > 30 ng/ml	CPR; Ongoing pregnancy rate	Low vitamin D level was not related to decreased CPR with egg donation.	BMI
Paffoni et al. 2014 [12]	Italy	Prospective cohort study	Long GnRH-a Flare-up GnRH-ant IVF	335	Prior to to initiation of COH	< 20 ng/ml; ≥20 ng/ml	CPR; Implantation rate	High vitamin D level was related to higher CPR	Race BMI
Polyzos et al. 2014 [13]	Belgium	Retrospective cohort study	Irrespective of the protocol Single, blastocyst IVF/ICSI	368	On the day of hCG administration	< 20 ng/ml; ≥20 ng/ml	Chemical PR CPR; Live birth rate (LBR)	Low vitamin D level was related to lower clinical pregnancy rate and live birth rate	Season of embryo transfer
Rudick et al. 2014 [11]	USA	Retrospective cohort study	Egg donation recipients	99	At the time of down-regulation	< 20 ng/ml; 20-30 ng/ml; > 30 ng/ml	CPR; LBR	Higher 25(OH)D levels associated with higher CPR and LBR	Recipient age Recipient BMI Embryos transferred(n, quality)
Franasiak et al. 2015 [18]	Canada	Retrospective cohort study	Long GnRH-a Flare-up GnRH-ant FET/ICSI	517	On the day of ovulation trigger	< 20 ng/ml; 20-30 ng/ml; > 30 ng/ml	CPR Ongoing PR	Vitamin D had no relationship with IVF outcomes	Age No. of embryo transferred Race

### Meta-analysis

**Fig. 2:**
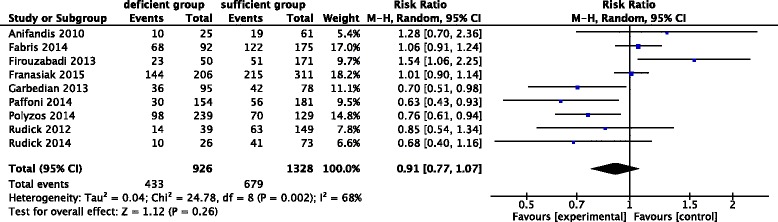
Forest plot showing the results of meta-analysis of studies comparing the effect of deficient vitamin D and sufficient vitamin D on clinical pregnancy after IVF/ICSI

The present meta-analysis included 9 studies to evaluate the association between deficient level vitamin D and the clinical pregnancy outcomes after ART treatments. We found that there was a trend (without significance) toward lower in clinical pregnancy in women with deficient level of vitamin D compared with those with sufficient level of vitamin D. As the Q statistic *P*-value was below 0.1, there were heterogeneity of the studies (I^2^ = 68%, *P* = 0.002). The random effects model was implied and the combined RR was 0.91 (95% CI, 0.77, 1.07; *P* = 0.26) (Fig. 2).

**Fig. 3:**
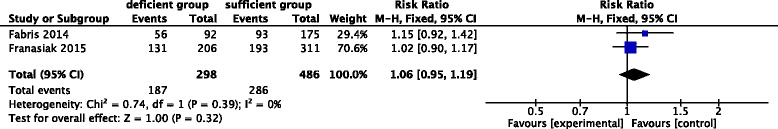
Forest plot showing the results of meta-analysis of studies comparing the effect of deficient vitamin D and sufficient vitamin D on ongoing pregnancy after IVF/ICSI

Among these studies, 2 papers evaluated the association between deficient vitamin D and on ongoing pregnancy at the same time. The results indicated that a similar ongoing pregnancy rate in patients with deficient and sufficient level vitamin D. The Q statistic *P*-value was above 0.1, indicating homogeneity of the studies (l^2^ = 0%, *P* = 0.39). The fixed effects model was implied and the combined RR was 1.06 (95% CI, 0.95, 1.19; *P* = 0.32) (Fig. 3).

**Fig. 4:**
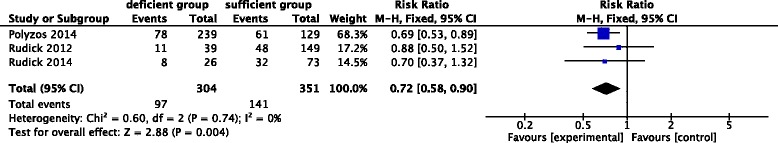
Forest plot showing the results of meta-analysis of studies comparing the effect of deficient vitamin D and sufficient vitamin D on live birth after IVF/ICSI

Additionally, live birth rate was also evaluated and 3 studies were included. The results of meta-analysis showed a significantly lower live birth rate in women with deficient level vitamin D compared with ones with sufficient level vitamin. There was little heterogeneity in the results (l^2^ = 0%, *P* = 0.74). The fixed effects model combined RR was 0.72 (95% CI, 0.58, 0.90; *P* = 0.004) (Fig. 4).

The studies got high score on the Newcastle-Ottawa Quality Assessment Scale (not shown). The funnel plots of meta-analysis evaluating the association between vitamin D levels and the ongoing pregnancy and live birth after IVF/ICSI showed symmetrical shapes, indicating no publication bias (Additional file 1: Figure S1 and Additional file 2: Figure S2). However, a modest publication bias was seen when the clinical pregnancy was evaluated (Additional file 3: Figure S3).

## Discussion

So far, only two systematic reviews and meta-analyses have evaluated the association between vitamin D level and the clinical outcomes of IVF/ICSI cycles. To our knowledge, the present study is the largest in regard to sample size with 3693 IVF/ICSI cycles. In the present review, 9 studies, 2 studies and 3 studies were included to evaluate the association between vitamin D and clinical pregnancy, ongoing pregnancy and live birth, respectively. Vitamin D level was statistically associated with live birth (RR 0.74; 95%CI 0.58–0.90) and was not related to clinical pregnancy (RR 0.91; 95% CI 0.77–1.07) and ongoing pregnancy (RR 1.06; 95% CI 0.95–1.19). The three RR value demonstrated that deficient vitamin D was associated with a decreased chance of live birth, while had similar probability in clinical pregnancy and ongoing pregnancy with women with sufficient vitamin D. The conclusion was agreement with the systematic reviews by Valeria et al. and Lv et al. [19, 20], but we included additional several publications and evaluated not only clinical pregnancy but also ongoing pregnancy and live birth after IVF/ICSI.

As mentioned above, Vitamin D plays an important role in the female reproductive system [3, 6]. Therefore, a woman’s vitamin D level is supposed to be related to reproductive health and pregnant outcomes. The effect of deficient vitamin D level on reproduction has been investigated since the 1970s. Some studies believed that deficient vitamin D might have detrimental effect on the women infertility and pregnant outcomes of IVF [7–10]. However, other studies found that vitamin D deficiency did not play a critical role in the outcome of ART [14–16]. These discrepancies may be attributed to differences in patients’ mean age, ethnicity, race, BMI, countries, social economic status, the season, exposure categorization, analysis methods, and study design [22].

Although the discrepancies between studies, the combined results indicated that vitamin D deficiency may bring negative effect on the outcome of ART. The possible mechanism may be as follows:

Firstly, it was reported that vitamin D might have effect on the development of follicle and embryo. Mice, in which the VDR genes have been knocked out, showed damaged folliculogenesis and underdevelopment of uterine in a previous animal experiments [23]. Vitamin D brought beneficial effect on the ovarian steroidogenesis and stimulated the production of Insulin-like growth factor-binding protein-1 (IGFBP-1) in ovary. Vitamin D promotes the production of estradiol, estrone, and progesterone [24]. Vitamin D level of reproductive age women was correlated with their serum AMH levels [25], so vitamin D may involved in the production of AMH in adults.

Secondly, Vitamin D was identified with influence upon endometrial receptivity. It was identified that vitamin D receptor was expressed in the endometrium of mice [26], whereas vitamin D receptor mutant female mice have an underdeveloped uterus and were infertile [27]. One study demonstrated the 1,25(OH) _2_D_3_ administration could up-regulate the expression of HOXA10 mRNA and protein by combining with its receptor in the endometrial stromal cells, and HOXA10 is essential for female fertility and embryo implantation [28]. This view was further supported by a vitro cell experiments with human endometrial cells lines, indicating that the 1a–hydroxylase enzyme was up-regulated in the endometrial stromal cells of early pregnancy [29]. In an oocyte donor study, adjusted clinical pregnancy rates and live birth rate were lower in VD-deficient recipients than those of vitamin D sufficient recipients. This study suggested that vitamin D might affect the reproduction through mediating the endometrium, not the follicular or oocytes [11].

Thirdly, recent researches demonstrated that vitamin D is significant not only for embryo implantation but also for gestation as well. Clinical studies have shown that pregnant women with deficient vitamin D are more likely to be with preeclampsia, gestational diabetes, and cesarean section [30–32]. Varying levels expression of vitamin D and HOXA10 were tested in the endometrium, decidua and placenta throughout pregnancy [33]. Therefore, it was suggested that vitamin D plays a crucial role in keeping healthy pregnancy [34]. These may be the possible explanations for higher ongoing pregnancy rate while lower live birth rate.

Additionally, vitamin D affects the implantation and early pregnancy by involving the immune-modulating effect. Some studies showed that vitamin D may inhibit the activity of decidual T-cell and reduce the production of some cytokines such as interleukin 1 (IL-1), IL-6 and TNF-a, which are considered to be essential for embryo implantation and endometrial receptivity [29, 35]. The vitamin leads to a transition from T helper 1(TH-1) to the more tolerant TH-2. Endometrial cells produce 1a hydroxylase, which activates 25-OH vitamin D and is up-regulated by IL-1β produced by the blastocyst. Lower level of vitamin D has a tendency to increase the percentage of B cells, INF-a producing Th cells and NK cytotoxicity, all of these were factors for RM [36].

With regard to strength, the present study leads to a more accurate evaluation with the pooled RRs rather than individual study. The pooled results of included studies indicated that deficient vitamin D was associated with decreased chance of live birth after IVF/ICSI cycles. While evaluating the association between vitamin D and clinical pregnancy and ongoing pregnancy, the combined RRs showed a decreased trend in clinical pregnancy [RR 0.91; 95% CI 0.77–1.07] and an increased trend in ongoing pregnancy [RR 1.06; 95% CI 0.94–1.19] but the differences had no significances.

Certainly, the present meta-analysis still has its limitations. A major limitation was the obvious heterogeneity among these included studies’ characteristics, including varied study designs (retrospective/retrospective studies), different ovarian stimulation protocols (GnRH-a long or short protocol/GnRH-ant protocol), different treatment types (IVF / ICSI) and different threshold for deficient vitamin D level (20 ng/ml, 10 ng/ml or 75 nmol/L). In addition, small sample size of some included studies and short of meaningful confounders adjustment were also the limitations of the present study. In spite of these shortcomings, the present systematic review and meta-analysis reviewed and summarized the results of relative publications with valuable summary.

## Conclusion

This systematic review showed that deficient vitamin D has negative effect on the pregnancy after IVF/ICSI treatment. As the sample sizes of some studies were small and the study characteristics were different, further random cohort studies are needed to observe the effect of vitamin D supplementation and explore the possible effect mechanisms.

## Additional files

Additional file 1: Figure S1. Funnel plot of analysis for the effect of vitamin D level on pregnancy, showing the results of Eggers to assess publication bias. (PDF 19 kb)

Additional file 2: Figure S2. Funnel plot of analysis for the effect of vitamin D level on ongoing pregnancy, showing the results of Eggers to assess publication bias. (PDF 17 kb)

Additional file 3: Figure S3. Funnel plot of analysis for the effect of vitamin D level on live birth, showing the results of Eggers to assess publication bias. (PDF 18 kb)

## Abbreviations

ART: Assisted reproductive technology
CI: Confidence interval
ICSI: Intracytoplasmic sperm injection
IR: Implantation rate
IVF: In vitro fertilization
OR: Odds ratio
PR: Pregnancy rate
