# Supplementary figures and images for: Whether vitamin D was associated with clinical outcome after IVF/ICSI: a systematic review and meta-analysis
Source: Reprod Biol Endocrinol. 2018 Feb 9;16:13. doi: 10.1186/s12958-018-0324-3 (PMC5807754; doi:10.1186/s12958-018-0324-3)

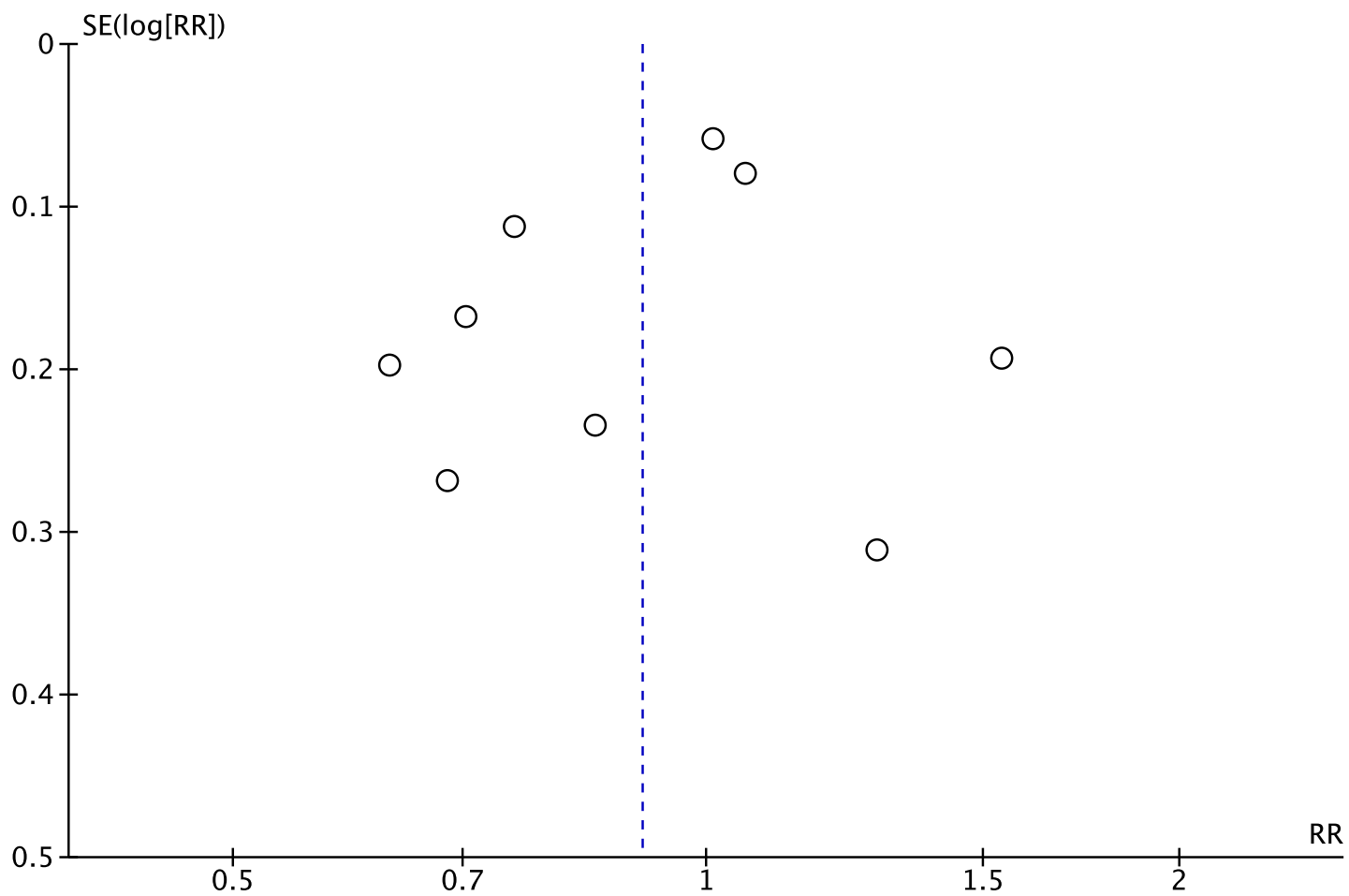

Supplement: Supplementary file 1 — Funnel plot of analysis for the effect of vitamin D level on pregnancy, showing the results of Eggers to assess publication bias. (PDF 19 kb) [file 12958_2018_324_MOESM1_ESM.pdf]

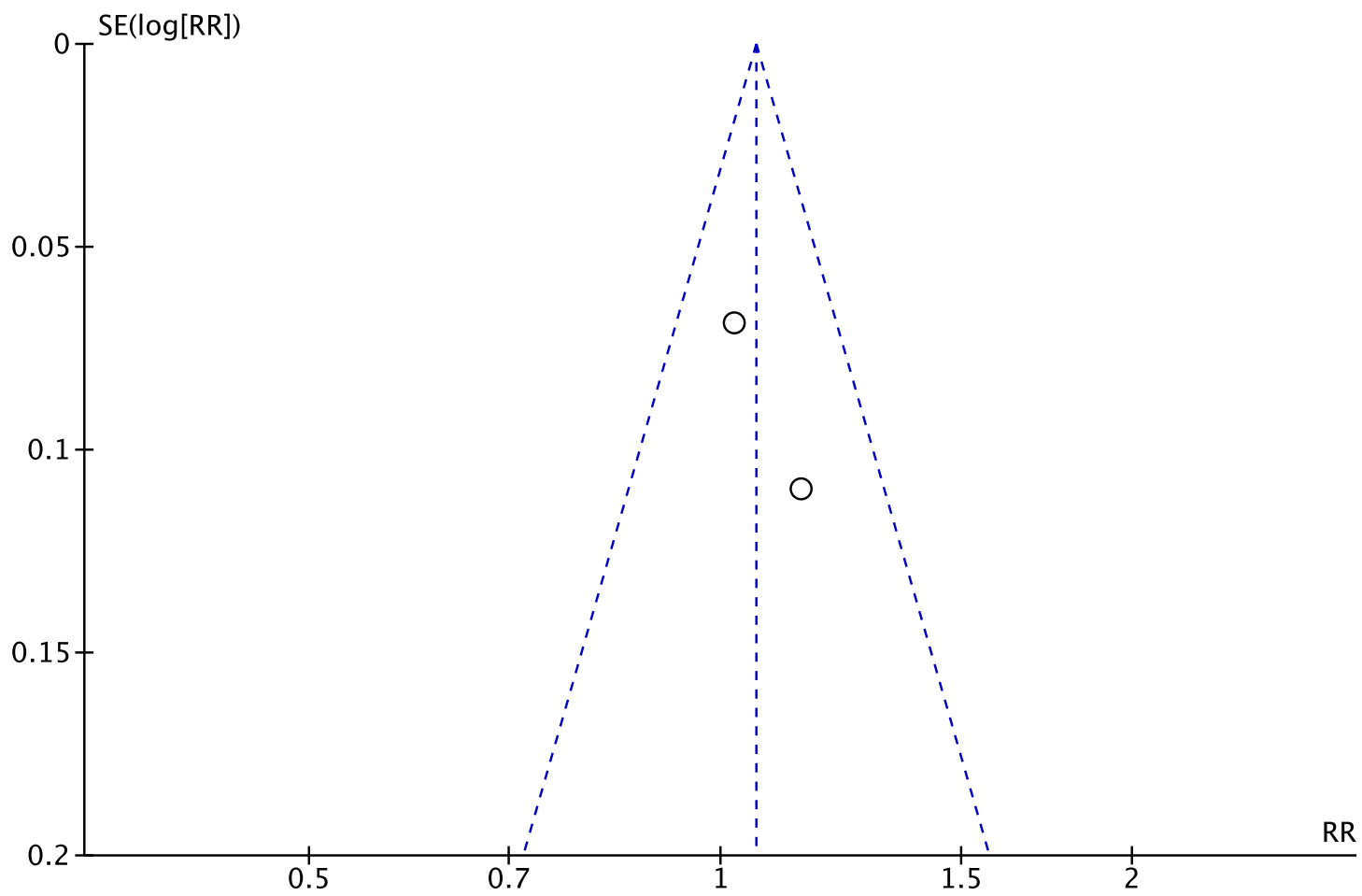

Supplement: Supplementary file 2 — Funnel plot of analysis for the effect of vitamin D level on ongoing pregnancy, showing the results of Eggers to assess publication bias. (PDF 17 kb) [file 12958_2018_324_MOESM2_ESM.pdf]

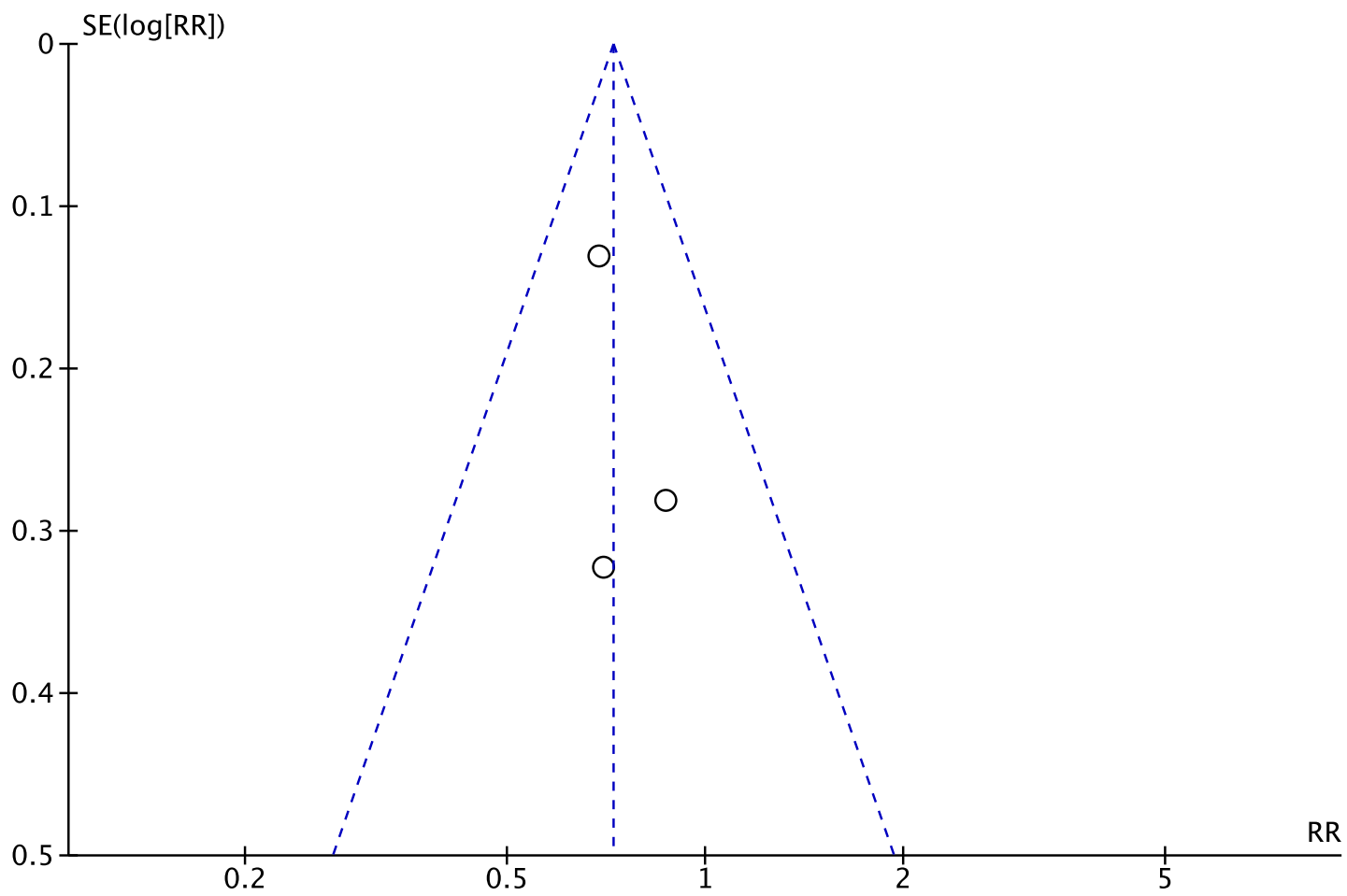

Supplement: Supplementary file 3 — Funnel plot of analysis for the effect of vitamin D level on live birth, showing the results of Eggers to assess publication bias. (PDF 18 kb) [file 12958_2018_324_MOESM3_ESM.pdf]
